# Unveiling the functional epitopes of cobra venom cytotoxin by immunoinformatics and epitope-omic analyses

**DOI:** 10.1038/s41598-023-39222-2

**Published:** 2023-07-28

**Authors:** Jia Jin Hiu, Jared Kah Yin Fung, Hock Siew Tan, Michelle Khai Khun Yap

**Affiliations:** School of Science, Monash University Malaysia, 47500 Bandar Sunway, Malaysia

**Keywords:** Protein sequence analyses, Mass spectrometry, Immunology

## Abstract

Approximate 70% of cobra venom is composed of cytotoxin (CTX), which is responsible for the dermonecrotic symptoms of cobra envenomation. However, CTX is generally low in immunogenicity, and the antivenom is ineffective in attenuating its in vivo toxicity. Furthermore, little is known about its epitope properties for empirical antivenom therapy. This study aimed to determine the epitope sequences of CTX using the immunoinformatic analyses and epitope-omics profiling. A conserved CTX was used in this study to determine its T-cell and B-cell epitope sequences using immunoinformatic tools and molecular docking simulation with different Human Leukocyte Antigens (HLAs). The potential T-cell and B-cell epitopes were 'KLVPLFY,' 'CPAGKNLCY,' 'MFMVSTPTK,' and 'DVCPKNSLL.' Molecular docking simulations disclosed that the HLA-B62 supertype exhibited the greatest binding affinity towards cobra venom cytotoxin. The namely L7, G18, K19, N20, M25, K33, V43, C44, K46, N47, and S48 of CTX exhibited prominent intermolecular interactions with HLA-B62. The multi-enzymatic-limited-digestion/liquid chromatography-mass spectrometry (MELD/LC–MS) also revealed three potential epitope sequences as 'LVPLFYK,' 'MFMVS,' and ‘TVPVKR’. From different epitope mapping approaches, we concluded four potential epitope sites of CTX as ‘KLVPLFYK’, ‘AGKNL’, ‘MFMVSTPKVPV’ and ‘DVCPKNSLL’. Site-directed mutagenesis of these epitopes confirmed their locations at the functional loops of CTX. These epitope sequences are crucial to CTX’s structural folding and cytotoxicity. The results concluded the epitopes that resided within the functional loops constituted potential targets to fabricate synthetic epitopes for CTX-targeted antivenom production.

## Introduction

The World Health Organization (WHO) has declared snakebite envenomation (SBE) as a category A Neglected Tropical Disease^1^. The cobra (*Naja* sp.) envenomation is unequivocally a feared snake species due to their cytotoxic venoms, leading causes of physical impairment that engenders lifetime handicaps in most survivors^2^. The cytotoxic envenomation is manifested as local dermonecrosis, which is a sequelae event due to cell death and subsequent loss-of-function injuries^3–6^. The venomics of *Naja* venom revealed that, the major venom toxins are phospholipase A_2s_ (PLA_2s_), neurotoxins (NTXs) and cytotoxins (CTXs). PLA_2s_ and CTXs constitute average 20% and 70% of the *Naja* venoms’ dry weight, respectively^7–10^. It is noteworthy that, synergisms of PLA_2s_ and CTXs have been summarized to cause downstream cytotoxic effects upon envenomation^11^. Nevertheless, CTXs play major roles in venom-induced dermonecrosis due to their abundances in *Naja* venom^12^.

Cytotoxins are highly basic and amphipathic polypeptides^13^. They are 7 kDa with approximately 59–62 amino acid residues^14^. They appear in three-finger loops from five antiparallel β-sheets^13^. These β-sheets make up the hydrophobic core of the toxins, which are stabilized by four conserved disulfide bridges^15^. The hydrophobic core is also flanked by basic lysine and arginine residues responsible for cytotoxicity^16^. It allows the toxins to interact with the cell membranes and contributes to the formation of membrane pores, resulting in subsequent cell death cascade events^17–20^. Thus, it is said that the CTX’s loops are responsible for dermonecrosis in snakebite victims.

Although SBE poses a significant global health risk, immunotherapy with heterologous antivenoms is the only currently effective intervention. Antivenoms are usually produced by isolating polyclonal antibodies or antibody fragments from the serum or plasma of immunized animals with venom^21^. However, they present undesirable drawbacks, especially exposing patients to the risk of hypersensitivity reactions and anaphylaxis^22,23^. Besides, the antivenoms exhibit limited neutralizing ability on the CTX-induced necrosis^12^. The low efficacy of the antivenoms suggests the inadequate immunogenicity of the CTXs to produce CTX-specific antibodies. Several factors affecting the immunogenic capability of CTXs including the molecular structure, and sequence variations^5,24^. Small molecular size of the toxins may contribute to the sparse antigenicity^25^. Moreover, CTXs exhibit low immunogenicity due to their three-finger folded structures that limit its surface exposure for epitope recognition. It is conspicuous that the existing antivenoms are ineffective against dermonecrosis due to their poor pharmacokinetic profiles^26^. This sheds light in the recruitment of biotechnological tools to expedite the development of next generation antivenom, which allows the immunological characterization of venom toxins responsible for envenomation^27,28^.

By characterizing the epitopes of venom toxins, the possible immunogenic sites of toxins can be identified to aids in developing appropriate antivenoms specifically targeting the respective toxins. Computational analysis has been reported to examine the immunogenicity of toxins, such as three-finger toxins (3FTX), phospholipase A_2_s (PLA_2s_), cysteine-rich secretory proteins (CRISP), disintegrins (DIS), Kunitz peptides (KUN), L-amino acid oxidases (LAAO), natriuretic peptides (NP), snake C-type lectins (SNACLEC), snake venom metalloproteinases (SVMP) and snake venom serine proteases (SVSP) ^25,29,30^. Furthermore, high-throughput approaches such as peptide microarray, mass spectrometry (MS) epitope mapping, and phage display methods have also been developed to characterize the toxin-antivenom interaction^29–33^.

Epitopes are short molecular regions of an antigen recognized by the immune system^34,35^. The recognition of epitopes elicits immune responses to produce antibodies that bind to eradicate the antigen and inhibit its toxic effects^36^. This is the fundamental principle in current antivenom production, which involves the neutralization of venom toxins by the antibody^37^. Recent research demonstrates innovative venom-independent immunization strategies using synthetic epitopes for antibody production^37^. Such epitopes have shown more direct and potent protectivity than the immune response conferred by whole protein^34,38^. Notably, most ongoing research today focuses on the whole venom instead of toxin-specific antitoxins of interest^39^. The understanding of immunogenic properties of venom toxins shows the prospects of producing standardized synthetic epitopes for specific and effective antivenoms without causing toxicity to the host.

Despite the promising prospects of synthetic epitopes of toxins, limited knowledge is known on utilizing synthetic epitopes in CTXs-targeted therapeutics. Furthermore, the functional epitopes properties of CTXs remain unclear. Therefore, in this study, epitope characteristics of CTXs were determined to identify the potential epitopes of significant relevance as synthetic epitope leads for next generation antivenoms. This study used a conserved CTX sequence deduced from various CTX isoforms from different *Naja* species, aiming to overcome the predicaments of species diversity and geographical variation^40^. We applied immunoinformatic and multi-enzymatic-limited-digestion/liquid chromatography-mass spectrometry (MELD/LC–MS) to determine the immunogenic properties of the conserved CTX can be determined. Furthermore, we performed site-directed mutagenesis to validate the presence of epitope sites at the hydrophobic functional loops of CTX.

## Results and discussions

### Epitope prediction

#### T-cell epitope prediction

The list of predicted T-cell epitopes is summarized in Table 1. A total of 15 potential T-cell epitopes associated with 9 major histocompatibility complex (MHC) supertypes were identified. Some of these epitopes showed sequence and length similarities despite being classified into different MHC supertypes.

**Table 1:** Summary of T-cell epitope sequence parameters across different MHC supertypes-frequency of epitope across supertypes, probability of epitope presence across supertypes, values of affinity supertype, TAP efficiency, average combined score, and sensitivity according to MHC supertypes ranking analysis.

T-cell epitope sequence	Functional Loop	MHC Supertypes	Frequency	Probability	Affinity supertype	TAP efficiency	Average combined score	Sensitivity
CPAGKNLCY	-	A1, B7	2	0.2222	0.0930(A1), 0.1297(B7)	2.606	0.5977	0.89
CPKNSLLVK	III	A3	1	0.1111	0.1864	0.164	0.5024	0.89
DVCPKNSLL	III	A26	1	0.1111	0.1719	0.1719	0.5584	0.89
FMVSTPTKV	II	A2	1	0.1111	0.7859	0.455	1.2922	0.54
FYKTCPAGK	-	A24	1	0.1111	0.2573	0.6583	0.68	0.89
GKNLCYKMF	-	B27	1	0.1111	0.1496	− 1.879	0.56	0.89
KLVPLFYKT	I	A2	1	0.1111	0.5875	− 0.36	0.9591	0.74
KMFMVSTPT	II	A2, A3	3	0.3333	0.5235(A2),0.2772(A3), 0.3642(B62)	− 0.417	0.6917	0.89
KRGCIDVCP	-	B27	1	0.1111	0.2023	0.5597	0.302	< 0.89
KTCPAGKNL	-	B58	1	0.1111	0.2239	0.997	0.653	0.89
MFMVSTPTK	II	A3, A24	2	0.2222	0.5555(A3), 0.2257(A24)	0.9191	0.845	0.8
NLCYKMFMV	-	A2	1	0.1111	0.5703	0.9341	0.481	< 0.89
NNKLVPLFY	I	A1, A26	3	0.3333	0.0824(A1), 0.1116(A26),0.1257(B62)	2.8	0.5792	0.89
STPTKVPVK	II	A3	1	0.1111	0.3455	0.8205	0.528	0.89
VPVKRGCID	-	B7	1	0.1111	0.4386	0.795	0.7625	0.8

The parameters such as frequency of epitope among supertypes, affinity ranking, transporter associated with antigen processing (TAP) efficiency, and combined score were analyzed for ranking statistics (Table 1). The identical epitope sequences were then grouped. The frequency of T-cell epitopes present among 9 MHC supertypes was determined using equation (1).

Equation (1):

$${\text{Frequency}}\;{\text{T-cell}}\;{\text{epitopes}} = \frac{{{\text{Frequency}}\;{\text{of}}\;{\text{epitope}}\;{\text{across}}\;{\text{supertypes}}}}{{{9}\;{\text{MHC}}\;{\text{supertypes}}}}$$

The frequency value determined which T-cell epitopes were predominant in the CTX. This can be deduced by ranking the T-cell epitopes according to their frequency of presence.

The sequences ‘NNKLVPLFY’ and ‘KMFMVSTPT’ exhibited the highest probability of 0.3333 across all MHC supertypes (Table 1). Both epitope sequences are made up of amino acid residues within the functional loops I and II of CTX, which are responsible for its cytotoxicity. The epitope sequences of ‘CPAGKNLCY’ and ‘MFMVSTPTK’ showed a probability of 0.2222, whereby ‘MFMVSTPTK’ shared 78% sequence homology to that of ‘KMFMVSTPT,’ which has a superior presence probability of 0.3333 with only discrepancy of an amino acid residue. The remaining predicted T-cell epitopes exhibited only probability values of 0.1111 across different MHC supertypes. CTX consists of three functional loops responsible for cytotoxicity^13,14,16^. Despite some predicted T-cell epitope sequences such as 'CPKNSVK' and 'DVCPKNSLL' resided entirely on the functional loops' amino acids, these sequences somehow demonstrated a lower presence probability value of 0.1111.

The affinity (supertype) scoring represents the predicted MHC binding affinity as -log_50000_(affinity score), whereby log_50000_ is the logarithm with base 50,000 for the affinity score. The higher the scoring, the higher the binding affinity. Based on the result, 'FMVSTPTKV' exhibited a higher affinity score (Table 1). This epitope sequence resided within the functional loop II of CTX. This was followed by 'KLVPLFYKT,' which resided on the functional loop I. Despite so, some epitopes on functional loops showed relatively lower affinity scores, for example, 'CPKNSLLVK,' 'DVCPKNSLL,' and 'NNKLVPLFY.'

The ranking analysis of the TAP efficiency of each predicted T-cell epitope sequence was also performed. The interactions of cytotoxic T-cells with other cells abide by a strict and specific recognition process dictated by T-cell epitopes presented on the surfaces of human leukocyte antigens (HLAs)^41^. However, before they are presented on the surfaces of HLAs, the transport associated with antigen processing (TAP) recognizes and transports these epitope sequences into the endoplasmic reticulum^42^. Therefore, the role of TAP must significantly influence the selection of T-cell epitopes. The TAP efficiency ranking showed that 'NNKLVPLFY' and 'CPAGKNLCY' exhibited TAP efficiency scores of 2.8000 and 2.6060, respectively. The third-ranked TAP efficiency sequence was 'KTCPAGKNL.' There was a drastic difference in TAP efficiency scores among the epitope sequences, presumably due to the strict specificity of TAP binding with peptide sequences. Notably, the amino acid residue 'KT' were found in the epitopes with lower TAP efficiency scores, indicating that these two amino acids significantly reduced the TAP efficiency. Structurally, these two amino acids comprise the β-sheet of loop I in the CTX. Their structural role may be accounting for their stringent non-interactive characteristics. Interestingly, none of the amino acid residues of 'CPAGKNLCY' was found to reside within the functional loops of CTX. This epitope sequence constituted the region that connected loops I and II of CTX, suggesting that this sequence may facilitate CTX-membrane interactions.

The next parameter used in predicting T-cell epitopes was combined score ranking. The combined score consists of an integrated peptide binding affinity, C-terminal cleavage affinity, and TAP efficiency scores, which are all significantly involved in the MHC Class I processing pathway^43^. To determine a compromise between sensitivity and specificity, a threshold of 0.75 for the combined score of predicted epitope sequences was used for selection. Thus, the predicted four epitope sequences 'FMVSTPTKV,' 'KLVPLFYKT,' 'MFMVSTPTK,' and 'VPVKRGCID' exhibited the highest chances as potential T-cell epitopes of CTX.

Based on the results, 'NNKLVPLFY' and 'KLVPLFYKT' were found within loop I, while 'FMVSTPTKV' and 'MFMVSTPTK' were situated in loop II. 'CPAGKNLCY' constituted the region between loops I and II of CTX. These sequences were the best sequences representing T-cell epitopes of CTX. Although 'CPAGKNLCY' was not situated in the functional loops of CTX, this epitope sequence exhibited a greater TAP efficiency score and presence probability among all MHC supertypes. Furthermore, this sequence was amphiphilic and highly basic, conserved in CTX for interaction with targets^13^. This epitope sequence also connected the two prominent β-sheets of loops I and II, which are vital to the overall functionality and stability of CTX. Since functional loops of CTX are important for cytotoxicity, and antivenoms have a binding affinity towards functional sites of toxins, it is feasible to deduce that the amino acid sequences that make up the functional loops were potential T-cell epitopes^14,28,44^.

#### B-cell epitope prediction

##### Protrusion index score

The protrusion index (PI) is a simple and effective concept for identifying protein regions that protrude from its surface. A high PI score is often related to sites of antigenicity^45^. The B-cell epitope prediction based on the PI score for CTX was summarized in Table 2. Four predicted linear B-cell epitopes were 'DVCPKNSLL,' 'MVSTPTKVP,' 'KLVPLFYKTCPAGKN,' and 'NTDRCN.' Of these B-cell epitopes, 'DVCPKNSLL,' 'MVSTPTKVP,' and 'KLVPLFYKTCPAGKN' were found on the functional loops of CTX. The residues within the functional loops were underlined in the predicted discontinuous B-cell epitopes.

**Table 2:** Prediction of linear and discontinuous B-cell epitope sequences by protrusion index score, underlined residues represent the residues found on the functional loops of cytotoxin.

Start	End	Linear B-cell epitope sequence	Number of residues	Protrusion index score
42	50	DVCPKNSLL	9	0.676
27	35	MVSTPTKVP	9	0.665
6	20	KLVPLFYKTCPAGKN	15	0.632
57	62	NTDRCN	6	0.524

Residues			Number of residues	Protrusion index score
$\underline{{{\text{N}}^{{\text{5}}} }} ,\;\underline{{{\text{K}}^{{\text{6}}} }} ,\;\underline{{{\text{L}}^{{\text{7}}} }} ,\;\underline{{{\text{V}}^{{\text{8}}} }} ,\;\underline{{{\text{P}}^{{\text{9}}} }} ,\;\underline{{{\text{L}}^{{{\text{1}}0}} }} ,\;\underline{{{\text{F}}^{{{\text{11}}}} }} ,\;\underline{{{\text{Y}}^{{{\text{12}}}} }}$			8	0.645
$$\begin{aligned} & {\text{L}}^{{\text{1}}} ,\underline{{{\text{K}}^{{\text{2}}} }} ,\underline{{{\text{T}}^{{{\text{14}}}} }} ,\underline{{{\text{C}}^{{{\text{15}}}} }} ,{\text{ P}}^{{{\text{16}}}} ,{\text{ A}}^{{{\text{17}}}} ,{\text{ G}}^{{{\text{18}}}} ,{\text{ K}}^{{{\text{19}}}} ,{\text{ N}}^{{{\text{2}}0}} ,\underline{{{\text{M}}^{{{\text{27}}}} }} ,\underline{{{\text{V}}^{{{\text{28}}}} }} ,\underline{{{\text{S}}^{{{\text{29}}}} }} , \\ & \underline{{{\text{T}}^{{{\text{3}}0}} }} ,\underline{{{\text{P}}^{{{\text{31}}}} }} ,\underline{{{\text{T}}^{{{\text{32}}}} }} ,\underline{{{\text{K}}^{{{\text{33}}}} }} ,\underline{{{\text{V}}^{{{\text{34}}}} }} ,\underline{{{\text{P}}^{{{\text{35}}}} }} ,\underline{{{\text{D}}^{{{\text{42}}}} }} ,{\text{V}}^{{{\text{43}}}} ,{\text{ C}}^{{{\text{44}}}} ,{\text{ P}}^{{{\text{45}}}} ,{\text{ K}}^{{{\text{46}}}} ,{\text{ N}}^{{{\text{47}}}} , \\ & {\text{S}}^{{{\text{48}}}} ,{\text{ L}}^{{{\text{49}}}} ,\underline{{{\text{L}}^{{{\text{5}}0}} }} {\text{, N}}^{{{\text{57}}}} ,{\text{ T}}^{{{\text{58}}}} ,{\text{ D}}^{{{\text{59}}}} ,{\text{ R}}^{{{\text{6}}0}} ,{\text{ C}}^{{{\text{61}}}} \\ \end{aligned}$$			32	0.634

##### Parker hydrophilicity prediction

The basis of utilizing hydrophilicity to predict epitopes is attributed to the rational nature of epitopes on the surface, and they are exposed to be potentially antigenic^46^. The Parker hydrophilicity prediction of CTX is illustrated in Fig. S1A (Supplementary File). It was found that the hydrophilicity peaks surpassed the threshold value around amino acid residues 4–6, 15–21, 30–44, and 55–59, which represented hydrophilic and potentially antigenic sequences of 'LKCNNKLVP,' 'YKTCPAGKNLCYK,' 'MVSTPTKVPVKRGCIDVCPKNSL' and 'KYCCNTDRCN,' respectively.

##### Kolaskar & Tongaonkar antigenicity prediction

Kolaskar & Tongaonkar antigenicity prediction analyzes the physiochemical properties and abundance of amino acids within CTX compared to B-cell epitope references. Higher antigenicity scores are associated with a higher potential to induce immune responses^47^. As illustrated in Fig. S1B, peaks were observed at positions 8–14, 37–38, 41–43, 46–47, and 50–57, which represented sequences 'NKLVPLFYKTCPA,' 'VPVKRGCI,' 'RGCIDVCPK,' 'VCPKNSLL' and 'NSLLVKYVCCNTDR' respectively.

##### Emini surface accessibility prediction

An essential criterion of a potent epitope is its accessibility to other molecules for interaction. Emini surface accessibility prediction was therefore used to analyze and evaluate the surface accessibility of the amino acid residues. The linear B-cell epitopes were predicted based on their surface accessibility characteristics. As shown in Fig. S1C, 'STPTKVPV' appeared to be the most prominent peak with high surface accessibility at positions 29–35.

##### Karplus & Schulz flexibility prediction

Flexibility in protein structures is a vital characteristic that influences a protein’s ability to perform binding, catalysis, and allosteric interactions^48^. Karplus & Schulz's flexibility prediction was used to analyze the structural flexibility contributed by individual amino acid residues of CTX. As shown in Fig. S1D, the flexible regions were observed at positions 4–7, 14–21, 30–39, 45–49, and 58, which represented the sequences 'LKCNNKLVPL,' 'FYKTCPAGKNLCYK,' 'MVSTPTKVPVKRGCID,' 'DVCPKNSLLVK' and 'CCNTDRC,' respectively.

#### Multiple sequence alignment of T-cell and B-cell epitopes

**Figure 1:**
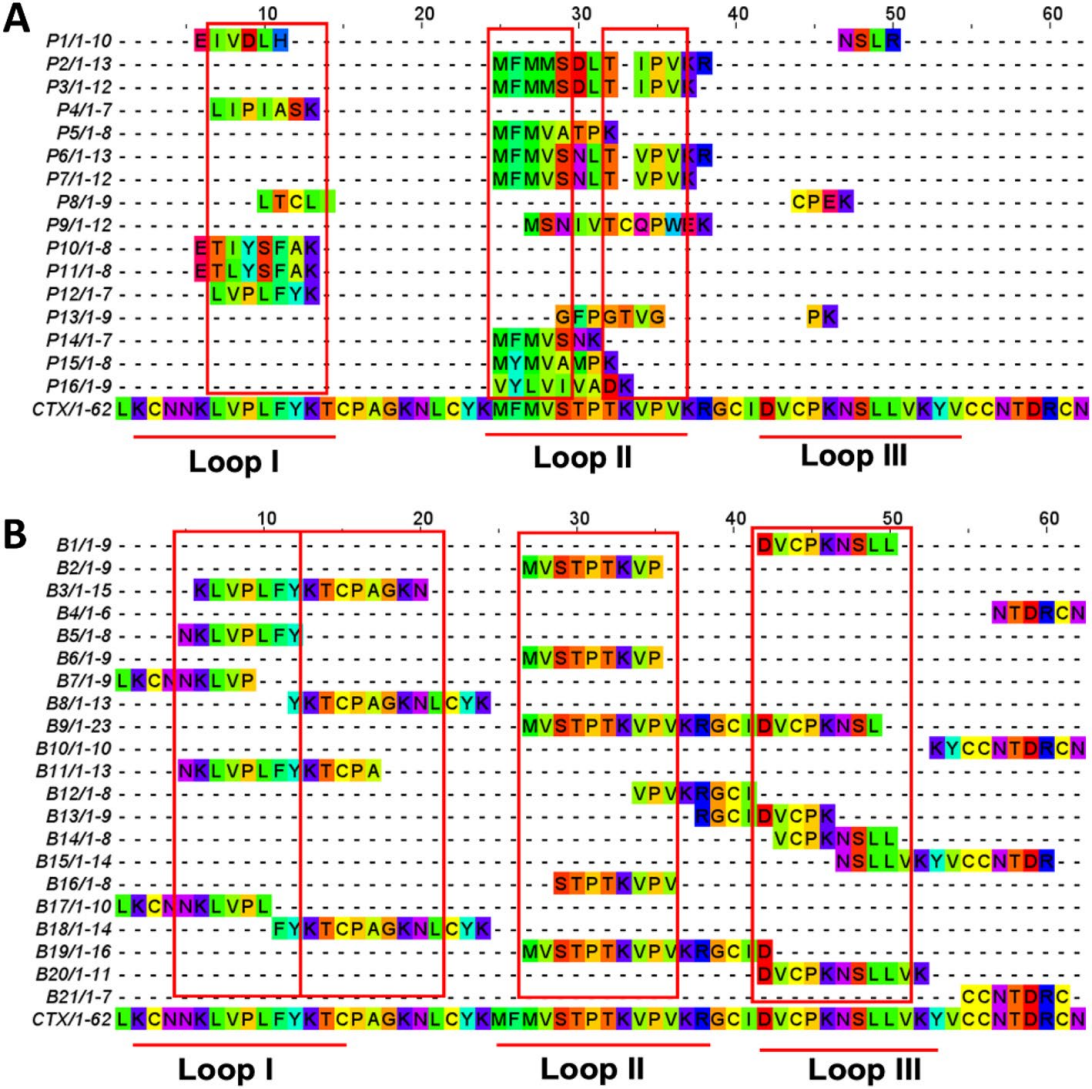
Multiple sequence alignment of predicted (**A**) T-cell epitopes, and (**B**) B-cell epitopes of cytotoxin. The red boxes represent the highly consensus residues.

The predicted T-cell and B-cell epitopes were aligned using Clustal Omega (https://www.ebi.ac.uk/Tools/msa/clustalo/)^49–51^. Based on the multiple sequence alignment, three potential T-cell epitope sequences of 'KLVPLFY,' 'CPAGKNLCY,' and 'MFMVSTPTK' were selected due to high consensus frequency (Fig. 1A). Of these sequences, 'KLVPLFY' and 'MFMVSTPTK' were found within the functional loops I and II, respectively. B-cell epitopes 'NKVLPLFY,' 'KTCPAGKNL,' 'MVSTPTKVP,' and 'DVCPKNSLL' were identified to have high consensus frequency (Fig. 1B). Interestingly, some of the identified T-cell and linear B-cell epitopes share significant sequence homology. For example, T-cell epitopes 'KLVPLFY,' 'CPAGKNLCY’ and ‘MFMVSTPTK’ shared similarities with B-cell epitopes ‘NKVLPLFY,' 'KTCPAGKNL,' 'MVSTPTKVP', respectively. Thus, four epitope sequences were concluded as potential epitopes for CTX. There were 'KLVPLFY,' 'CPAGKNLCY,' 'MFMVSTPTKVP,' and 'DVCPKNSLL.'

#### Molecular docking and molecular dynamic simulations of cytotoxin with HLA supertypes

The HADDOCK 2.2 web server clustered 583 structures into 89 clusters, representing 36.44% of the water-refined models. The docking parameters are summarized in Table 3. The HADDOCK score represents the weighted sum of all energies (*Van der Waals*, electrostatic interactions, desolvation energy, buried surface area, and restraints violation energy) between the docking structures. This scoring parameter was used to rank the docking clusters^52^. A lower HADDOCK score indicates a more reliable docking structure with better energetic stability^53,54^. HLA-B62 possessed the lowest HADDOCK score of 257.6 ± 30.1, followed by HLA-B27 and HLA-A02 (Table 3). The Z-score is a scoring function that predicts the binding affinity of multiple ligands towards a receptor, whereby a more negative Z-score indicates significant binding affinity^55^. All HLA-CTX complexes had negative Z-scores, but HLA-B07 and HLA-B62 had Z-scores of -1.9 and -2.0, respectively. This result suggested that HLA-B07 and HLA-B62 had the best binding affinity towards the T-cell and B-cell epitopes CTX. In molecular docking, the root-mean-square deviation (RMSD) value is used to assess whether a docked simulation between a ligand and receptor in a specific binding orientation and conformation are reproducible and values of < 2.0 Å indicate successful and appropriate docking^56^. Only HLA-B07 and HLA-B62 exhibited RMSD values < 2.0 Å, thus implying that they are reliable and reproducible docking structures. Therefore, HLA-B62 exhibited the best binding affinity and stability towards CTX's epitopes.

**Table 3:** Molecular docking interaction of cytotoxin with different HLA supertypes.

HLA supertypes	PDB ID	Best cluster	HADDOCK score	Z-score	RMSD value	Van der Waals (kJ/mol)	Electrostatic energy (KJ/mol)	Restraints violation energy (KJ/mol)	Desolvation energy (KJ/mol)
The docking parameters of cytotoxin with different HLA supertypes by molecular docking on HADDOCK2.2									
HLA-A01	3BO8	11	298.6 ± 21.3	− 0.5	7.7 ± 0.0	− 95.6 ± 16.2	− 603.5 ± 40.0	5145.4 ± 151.15	0.3 ± 5.4
HLA-A02	1AO7	11	266.1 ± 21.2	− 1.1	2.4 ± 0.2	122.9 ± 6.7	− 629.3 ± 40.6	5012.8 ± 150.34	13.6 ± 3.3
HLA-A03	3RL1	1	311.0 ± 18.3	− 1.4	7.2 ± 0.1	− 89.5 ± 9.8	− 482.8 ± 69.3	5010.5 ± 96.01	− 4.0 ± 6.2
HLA-A24	4F7P	4	331.7 ± 12.4	− 1.5	5.6 ± 0.2	− 85.1 ± 11.6	− 463.4 ± 71.0	5004.9 ± 342.98	9.0 ± 8.1
HLA-B07	5EO1	6	298.9 ± 49.8	− 1.9	1.2 ± 0.7	− 91.8 ± 14.1	− 576.8 ± 35.0	4890.7 ± 303.84	17.0 ± 9.3
HLA-B27	2BST	2	263.1 ± 15.1	− 1.5	8.4 ± 0.0	− 91.7 ± 7.4	− 616.5 ± 62.9	4579.8 ± 184.31	20.2 ± 9.5
HLA-B58	5IM7	14	270.1 ± 26.6	− 0.7	5.9 ± 0.2	− 125.0 ± 3.8	− 612.9 ± 103.5	5001.4 ± 124.67	17.5 ± 5.7
HLA-B62	3C9N	17	257.6 ± 30.1	− 2	1.1 ± 0.7	− 120.2 ± 12.7	− 676.5 ± 36.5	4971.9 ± 182.97	15.8 ± 3.4

HLA supertypes	Type of intermolecular interactions between HLA and cytotoxin (no. and %)					% Bond interacting with T-cell epitopes		% Bond interacting with B-cell epitopes	
	H-bond	Salt bridge	*Van der Waals*	π-π stacking	Cation-π				
Summary of intermolecular interactions between cytotoxin and HLA supertypes									
HLA-A01	21 (40%)	5 (9%)	26 (49%)	1 (2%)	0 (0%)	41.51		0	
HLA-A02	19 (25%)	3 (4%)	55 (71%)	0 (0%)	0 (0%)	42.86		24.86	
HLA-A03	24 (29%)	3 (4%)	54 (67%)	0 (0%)	0 (0%)	55.56		17.28	
HLA-A24	19 (32%)	2 (3%)	37(63%)	0 (0%)	1 (2%)	50.85		25.42	
HLA-B07	17 (29%)	3 (5%)	38 (66%)	0 (0%)	0 (0%)	43.01		17.24	
HLA-B27	24 (32%)	5 (7%)	43 (58%)	2 (3%)	0 (0%)	60		0	
HLA-B58	26 (30%)	5 (5%)	57 (65%)	0 (0%)	0 (0%)	43.18		21.59	
HLA-B62	19 (26%)	5 (5%)	48 (67%)	0 (0%)	0 (0%)	43.06		33.33	

**Figure 2:**
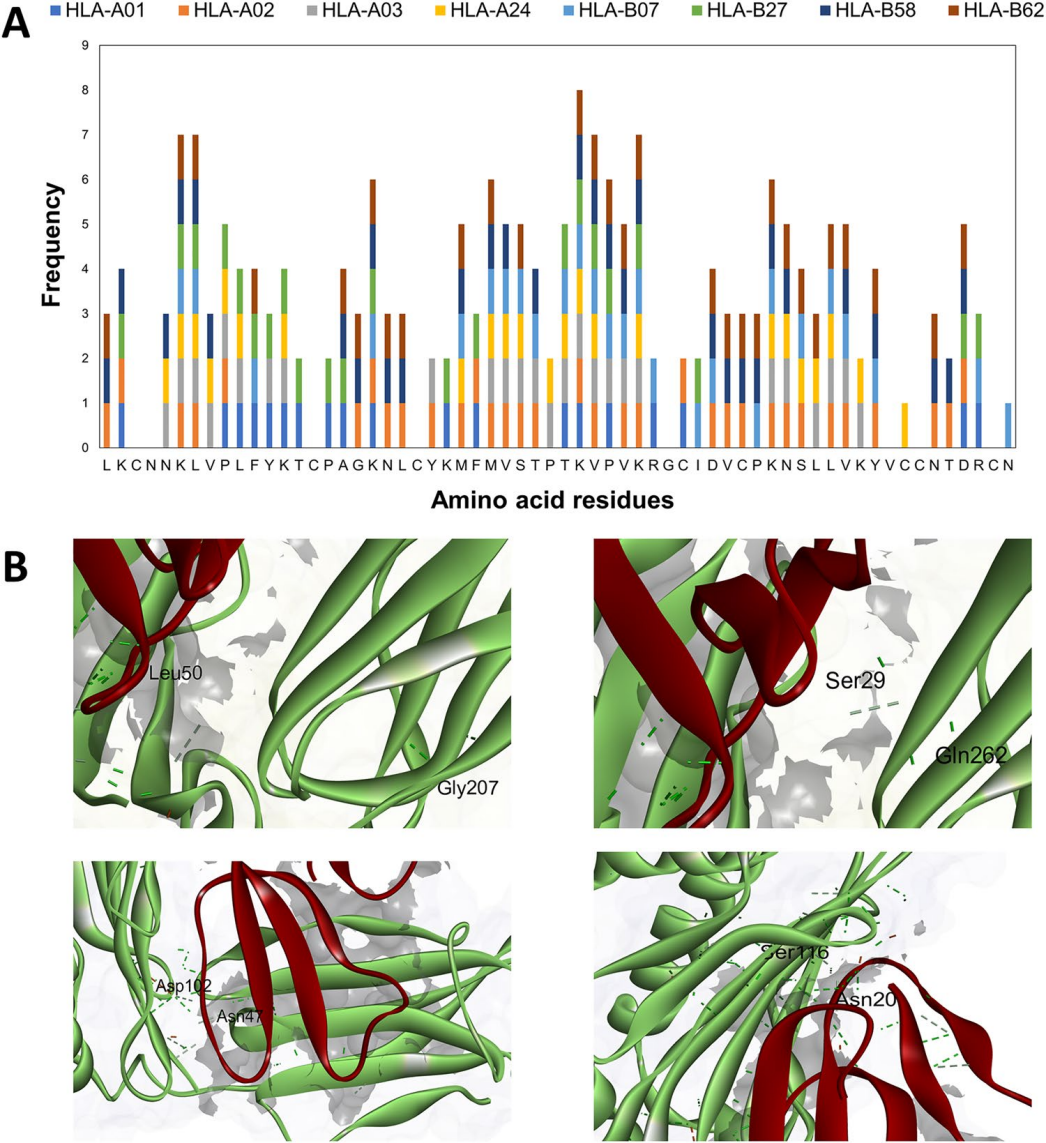
The molecular docking analysis of cytotoxin (CTX) with different Human Leukocyte Antigen (HLA) supertypes. (**A**) RING analysis for determination of interacting residues between CTX and eight HLA supertypes. Four regions indicated the highly frequent interacting sites (frequency ⩾3) suggesting these interacting sites were the potential epitopes. (**B**) Examples of interaction between CTX (red color) and the best docked HLA, HLA-B62 (green). The interacting residues were annotated, the dashed lines indicated hydrogen bonds.

Furthermore, we also performed Residue Interaction Network Generator (RING) analysis to determine the interacting residues between CTX and different HLA supertypes. RING analysis enables the identification of non-covalent interactions at the atomic level of a protein^57^. The RING analysis then revealed four highly interacting regions of 'NKLVPLFYK,' 'AGKNL,' 'MFMVSTPTLVPVK,' and 'DVCPKNSLLV' as the potential epitope sites, within the functional loops of CTX (Fig. 2A).

The significant intermolecular interactions within HLA-CTX complexes were *Van der Waals* (VDW) forces, hydrogen bonds, and salt bridges (Table 3). In comparison, π-π stacking and cation-π interaction were only observed in the docking structures of HLA-A01, HLA-A24, and HLA-B27. We considered the composition of the bonds within the docking complex to select the best HLA-CTX. Furthermore, a balance between the percentage of interacting bonds at the T- and B-cell epitopes was also considered. The π-π stacking and cation-π interactions were excluded as there is still limited information that serves as a reference for the best complex selection in docking simulations. The number of salt bridges was used as the first reference point for this selection because they constitute the most substantial interaction among all non-covalent molecular bonds. Among all the HLA-CTX complexes, HLA-A01, HLA-B27, HLA-B58, and HLA-B62 contained higher numbers of salt bridges with CTX's epitopes (Table 3). HLA-A01 and HLA-B27 were excluded because they did not interact with B-cell epitopes. By comparing HLA-B58 and HLA-B62, HLA-B62 had a relatively higher percentage of interactions with T-cell and B-cell epitopes (Fig. 2B), suggesting that HLA-B62 was the best binding HLA supertype with CTX.

The stability of the HLA-B62-CTX complex was further examined using molecular dynamic (MD) simulations) in a water box for 150 ns at 300 K. The mean RMSD value (Supplementary File-Fig. S2 was determined for the docking complex as 8.73 ± 0.80 Å. The mean MMPBSA potential energy of the docking complex remained at -23.55 ± 9.71 kcal/mol, suggesting the complex was stable during the simulation period. The mean number of hydrogen bonds of 1.56 ± 0.99 indicated the formation of hydrogen bonds within the HLA-CTX complex during simulation.

### Epitope-omic: MELD/LC–MS

The multi-enzymatic limited digestion (MELD) approach was first introduced by Morsa et al*.*^58^, which involves a cocktail of multiple diluted enzymes to generate peptide fragments. The synergistic effect of multi-enzymatic digestion results in enhanced sequence coverage and promising protein characterization^58^. This was due to the ability of the multi-enzymatic schemes to overcome the limitations of the mono-enzymatic digestion method, such as sequence-dependent digestion efficiencies and insufficient sequence coverage^58^. Therefore, the MELD/MS-based epitope mapping was employed to validate CTX’s epitopes. The epitope excision method was applied to exhibit no preference for either the “consecutive” or “assembled” epitopes^59^. This approach is based on the principle of specific binding between the antibody and antigen.

**Figure 3:**
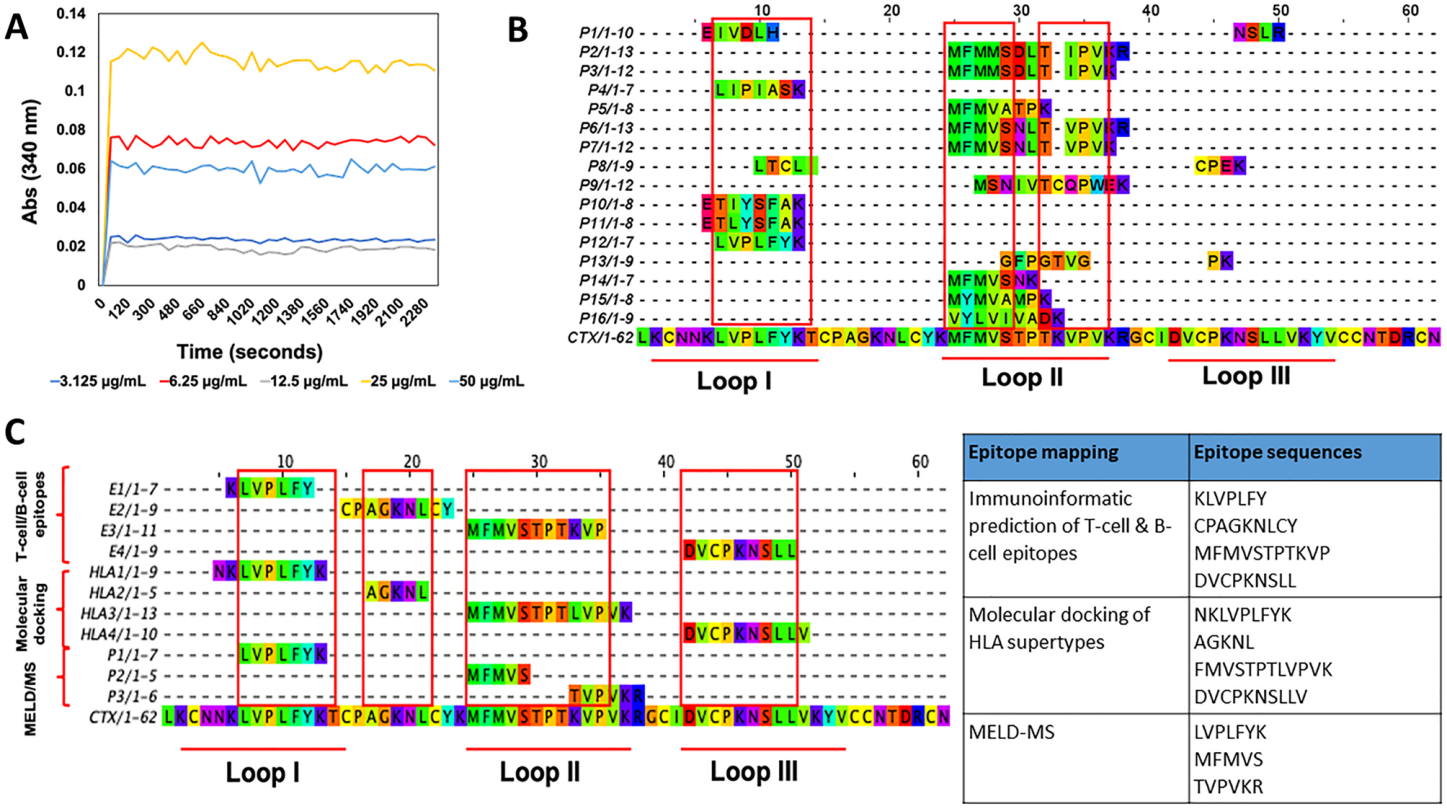
The alignment of epitope sequences determined by different epitope mapping approaches. (**A**) Immunocomplex kinetic analysis of CTX and anti-CTX, different concentrations of anti-CTX (3.125–50 µg/mL) were incubated with 12.5 µg/mL of CTX. Absorbance readings were measured at the wavelength (λ) of 340 nm (*n* = 3). (**B**) Multiple sequence alignment of MELD/LC–MS peptides with CTX revealed the presence of highly consensus sequences, annotated in red boxes. (**C**) Multiple sequence alignment and summary of epitope sequences determined from different mapping methods.

In this experiment, an anti-CTX monoclonal antibody with broad specificity was used to map the epitope sites on CTX. Upon binding to the anti-CTX, the CTX's epitopes were shielded from enzymatic proteolysis, and the exposed regions were digested. The reaction mixture was then subjected to LC–MS to identify the peptide(s) of the undigested region(s) known to be the epitopes. Prior to the MS-based epitope mapping, the immunoturbidimetric assay confirmed the formation of immunocomplexes between the anti-CTX and CTX. Based on the kinetic analysis (Fig. 3A), the formation of immunocomplexes peaked within the first minute and plateaued after that. The increased in absorbance reading reflects the turbidity formation. This is in corroborate with rapid formation of immunocomplexes as reported by O’Leary et al*.*^60^. The formation of immunocomplexes occurred from 3.125 to 50 µg/mL of anti-CTX, whereby the greatest turbidity was measured at 25 µg/mL of anti-CTX.

From the MELD/LC–MS results, 1:5000 diluted anti-CTX demonstrated a greater coverage of peptides mapping (Supplementary File-Table S1; Fig. S4). Thus, these identified peptide sequences were mapped to CTX (Fig. 3B). The multiple sequence alignments of LC–MS identified peptide sequences revealed three highly consensus sequences, ' LVPLFYK,' 'MFMVS,' and 'TVPVKR,' which were situated at the functional loops I and II of CTX (Fig. 3B).

In summary the epitope sequences of CTX determined from different mapping approaches (Fig. 3C) were finalized as ‘KLVPLFYK’, ‘AGKNL’, ‘MFMVSTPKVPV’ and ‘DVCPKNSLL’. Three of these sequences were located at the functional loops I, II, and III of CTX.

### Site-directed mutagenesis and cytotoxicity assay

Given that the epitopes ‘KLVPLFYK’, ‘MFMVSTPKVPV’ and ‘DVCPKNSLL’ were situated at functional loop I–III of CTX- (Fig. 3C), we performed site-directed mutagenesis to further confirm the presence of epitopes at functional loops. The membrane interactions of CTX^WT^ and CTX^VAR^ were expected to vary, which subsequently affected their cytotoxicity.

**Figure 4:**
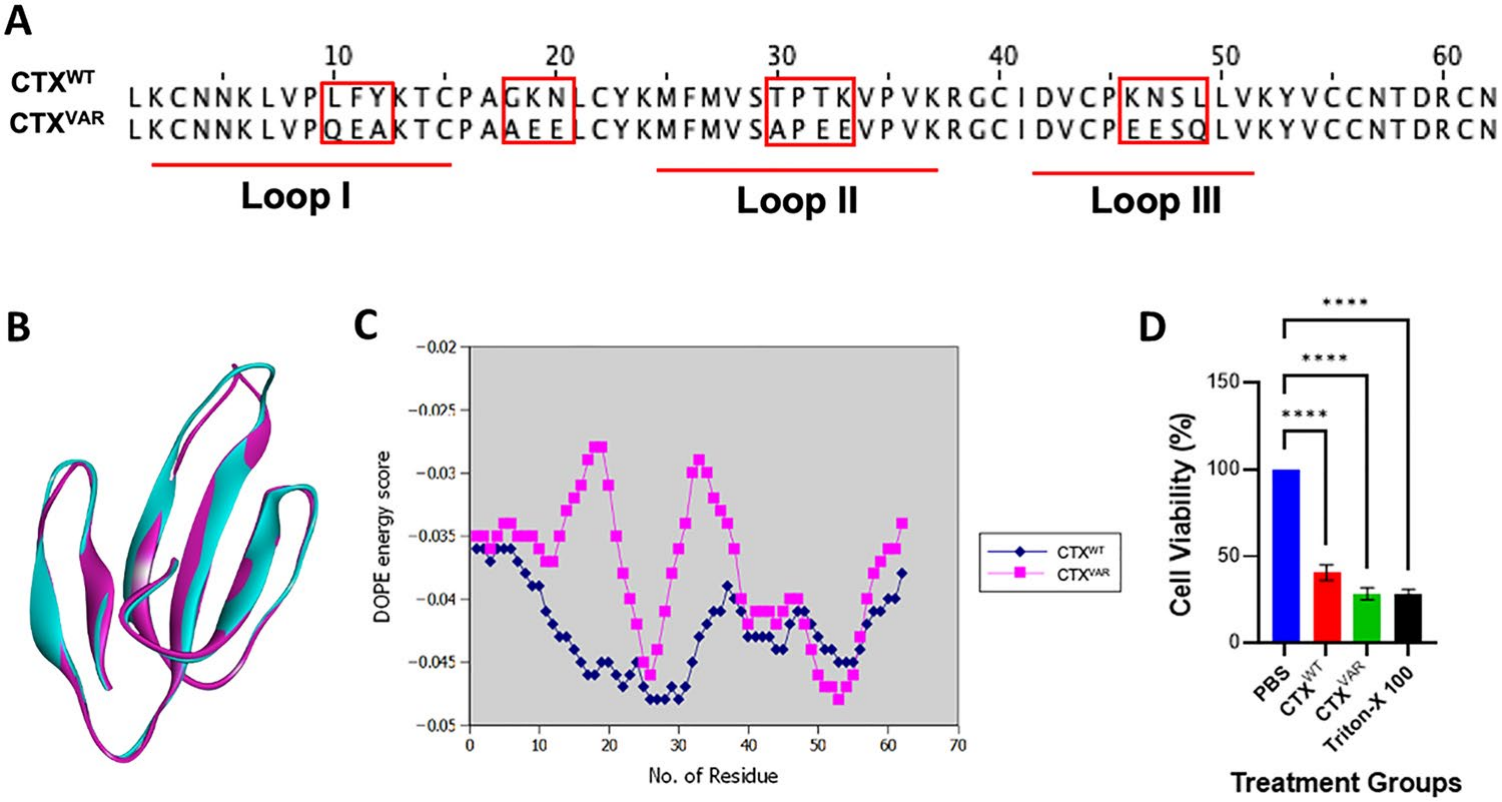
Site-directed mutagenesis analysis of cytotoxin (CTX) to determine its functionality. (**A**) Comparison of amino acid sequences between wildtype CTX (CTX^WT^) and variant (CTX^VAR^), red boxes indicate the mutagenesis sites. (**B**) Superimposed structural view of CTX^WT^ (cyan color) and CTX^VAR^ (purple color) showed similar three-finger folded structure. (**C**) DOPE per residue energy score against amino acid residue, CTX^VAR^ demonstrated variations at amino acid residues 10–12, 17–20, 30–34, and 46–49 compared to CTX^WT^. (**D**) Cell viability of HaCat cell lines after treatment with CTX^WT^ and CTX^VAR^ for 24 h. PBS and Triton-X 100 were used as negative and positive controls, respectively. The cytotoxic effect of CTX^VAR^ was slightly higher than the CTX^WT^; treatment groups. The data are shown as mean ± SEMs of three independent experiments (*n* = 3), the data was analyzed using one-way ANOVA whereby **** indicates *p* < 0.0001.

A CTX variant (CTX^VAR^) contained four mutated sites within the potential epitope sequences on the functional loops. The gene sequences of CTX^WT^ and CTX^VAR^ are as follows, underlined residues indicate mutated regions while asterisk (*) represents identical residues:
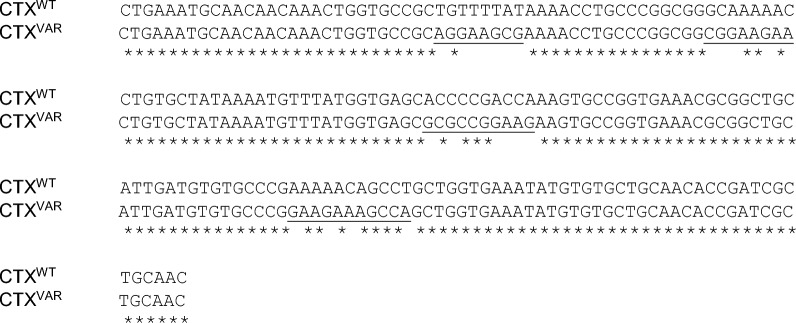


These mutated residues (Fig. 4A) were introduced based on the fact that the changes in amino acids did not affect the three-fingered structure of CTX (Fig. 4B). The RMSD values of both models were < 2.0 Å, indicating that they were all of good docking quality and did not deviate from the ideal orientation^61^. Nevertheless, the DOPE energy score (Fig. 4C) of CTX^VAR^ somehow showed variations at amino acids position 10–12, 17–20, 30–34, and 46–49 compared to CTX^WT^. These positions represented the four mutated sites that reside in the functional loops of CTX, whereby substantial variation was observed at functional loops I and II, which are vital towards the formation of the cytotoxin's hydrophobic core and cytotoxin-membrane binding motif^13^.

The alteration of amino acid residues in CTX^VAR^ somewhat changed its pI value, whereby CTX^VAR^ was more acidic (pI: 5.29) than CTX wildtype, CTX^WT^ (pI: 9.37). The interaction between CTX and the cell membrane is known to be closely related to the types of CTX, namely S-type and P-type. The insertion of S-type CTX into the cell membrane was primarily relying on the structural adaptation of loop II^62^. On the other hand, loops II and III of the P-type CTX were found to be more critical in lipid membrane penetration^63^. Additionally, studies showed that loop I of both S- and P-type CTX was involved in the membrane interaction^64,65^. Altogether, no conclusive finding supports a more significant loop among the three functional loops of the CTX during membrane penetration. However, loop II has been reported to involve in the membrane insertion for both S- and P-type CTX, implying that it plays a prominent role in the interaction between the CTX and cellular membranes.

The cytotoxicity of CTX^WT^ and CTX^VAR^ was determined in the human skin keratinocytes (HaCat cell lines). Both CTX^WT^ and CTX^VAR^ significantly (*p* < 0.0001) reduced the viability of the HaCat cell lines (Fig. 4D). It was observed that the CTX^VAR^ showed higher cytotoxicity than the CTX^WT^. The alteration of CTX^WT^ at the functional loops produced a more acidic CTX^VAR^ with enhanced cytotoxicity. Following the interaction of acidic CTX with cell membrane, it increased the acidity of cell membrane proteins to rigidify the membrane by expulsing water molecules, which ultimately shifts the cellular membrane to a gel phase^66^. Dehydration of the cellular membrane also influences dynamic membrane heterogeneity and facilitates membrane fusion, resulting in compromised membrane fluidity that leads to cell death^67,68^. The alteration of functional loops containing epitope sequences thus affected the cytotoxicity of CTX. The results confirmed the presence of epitope sequences at functional loops of CTX.

## Materials and methods

### Cytotoxin sequence

A conserved sequence of cytotoxin (CTX) was constructed in our laboratory^40^ based on consensus sequence obtained from multiple sequence alignment of the non-redundant and mature CTX sequences (NCBI Serpentes protein database, Taxid: 8570). These sequences constitute different CTX isoforms of different *Naja* species^40^. The final consensus CTX sequence is shown below:

$$\begin{aligned} & > {\text{CTX}} \\ & {\text{L}}\mathop {\underline{{{\text{KCNNKLVPLFYKTC}}}}}\limits_{{{\text{Loop I}}}} {\text{PAGKNLCY}}\mathop {\underline{{{\text{KMFMVSTPTKVPV}}}}}\limits_{{{\text{Loop II}}}} {\text{KRGCI}}\mathop {\underline{{{\text{DVCPKNSLLV}}}}} \limits_{{{\text{Loop III}}}} {\text{KYVCCNTDRCN}} \\ \end{aligned}$$

This conserved CTX is highly matched to *Naja atra* cytotoxin (accession no: Q98959.1, Taiwan origin) with 95% sequence similarity^40^.

### Epitope prediction

#### Prediction of T-cell epitope

T-cell epitope (CTL) prediction of the cobra venom cytotoxin was performed using the NetCTL 1.2 webserver (https://services.healthtech.dtu.dk/service.php?NetCTL-1.2) ^43^. The HLA supertypes selected for the prediction were A1, A2, A3, A24, A26, B7, B8, B27, B39, B44, B58, and B62; the threshold score for epitope identification was set at 0.50, while the weight on C terminal cleavage and TAP transport efficiency were 0.15 and 0.05, respectively. For the HLA-B62 supertype, a prototypical member of the supertype, HLAB*05:01, was used to represent it^69^. Statistical analyses were performed on each T-cell epitope candidate and ranked by four categories: frequency of epitope presence across supertypes, affinity score, transport associated with antigen processing (TAP) efficiency score, and combined score.

#### Prediction of B-cell epitope

The potential linear B-cell epitopes of CTX were identified using four different prediction algorithms, namely BepiPred-2.0 (https://services.healthtech.dtu.dk/services/BepiPred-2.0/) ^70^, IEDB analysis resource (http://tools.immuneepitope.org/bcell/) ^71^, antigenic peptide prediction method (http://imed.med.ucm.es/Tools/antigenic.pl) ^72^ and ABCPred (http://www.imtech.res.in/raghava/abcpred/) ^73,74^. Whereas the discontinuous B-cell epitopes were predicted using ElliPro webserver (http://tools.iedb.org/ellipro/) ^75^, provided by the Immune Epitope Database (IEDB) based on each amino acid residues' protrusion index score. Karplus & Schulz flexibility prediction (window size value of 7 and threshold value of 0.986), Kolaskar & Tongaonkar antigenicity (window size value of 7 and threshold value of 1.091), and Parker hydrophilicity prediction (window size value of 7 and threshold value of 0.895), was applied for the prediction. The predicted sequences from the outputs were aligned, whereby amino acid sequences with more than three overlapping frequencies were potential B-cell epitopes.

#### Human leukocyte antigen (HLA) distribution analysis

The T-cell epitopes (CTL) were also used for the HLA distribution analysis as the epitopes were also MHC ligands. The binding prediction of the epitopes to MHC Class I was evaluated using the peptide binding to MHC Class I molecules tool from the Immune Epitope Database (IEDB; http://tools.iedb.org/mhci/). NetMHCpan EL 4.1 was selected as the prediction method recommended by the IEDB for the binding prediction analysis across all alleles^76^. The peptide length was set as nonamer. The peptide-MHC I combinations with the highest scoring were filtered out from each peptide sequence input. A higher score indicates a higher affinity for the interaction between the peptide and MHC molecules. Prediction of the T-helper cell (HTL) epitopes for the CTX was performed using the peptide binding to MHC class II molecules tool from IEDB (http://tools.iedb.org/mhcii/). IEDB recommended NetMHCIIpan 4.1 EL was selected as the prediction method using specific MHC II alleles^76^. A 15-mer peptide length was selected. The peptide-MHC II interactions with the highest percentile rank in each set, indicating the strongest binding affinity, were filtered out for further analyses.

#### Molecular docking of cytotoxin with human leukocyte antigens (HLAs)

According to the predicted T-cell epitopes list, molecular docking analysis of T-cell epitopes with different HLA supertypes was performed on HADDOCK 2.2 to determine the interaction^77,78^. The PDB structures of different HLA supertypes were obtained from the RCSB PDB database. These HLA supertypes were HLA-A01 (3BO8), HLA-A02 (1AO7), HLA-A03 (3RL1), HLA-A24 (4F7P), HLA-B07 (5EO1), HLA-B27 (2BST), HLA-B58 (5IM7) and HLA-B62 (3C9N). CTX was set as the first molecule with functional loops' residues (residues 4–12, 27–36, 41–51) as active residues. The second molecule input was HLA supertypes, and all amino acid residues were set as active residues. The best docking clusters were determined by assessing each structure's HADDOCK score, Z-score, and RMSD value. Apart from that, the chosen docking cluster was accepted if the cluster maintained its native conformation, i.e., β-strands that are responsible for the stability of the structure. Later, the PDB of the best docking clusters was subjected to Residue Interaction Network Generator 2.0.1 (RING 2.0.1) analysis (http://old.protein.bio.unipd.it/ring/) ^57^ to determine the types and strength of both intra- and inter- non-covalent interactions of the docked structural clusters. The intermolecular non-covalent interactions between amino acids of CTX and HLA supertypes were mapped based on the aligned T-cell and B-cell epitopes.

#### Molecular dynamic (MD) simulations

The stability of CTX-HLA supertype complexes was further examined by molecular dynamic (MD) simulations using the GROMACS simulation package^79,80^. The MD simulations of all complexes were carried out for 150 ns in a solvated TIP3P water box using CHARMM 36 m force field, whereby the trajectory and energy files were written every 10 ps. Three chloride ions were added to the complex to neutralize the overall system. The protonation states were checked at pH 7 using the H +  + web server for His, Lys, Arg, Asp, and Glu residues. Minimization was carried out for 5000 steps using Steepest Descent Method, and the convergence was achieved within the maximum force < 1000 (KJ mol-1 nm-1) to remove any steric clashes. All three systems were equilibrated at NVT and NPT ensembles for 100 ps (50,000 steps) and 1000 ps (1,000,000 steps), respectively, using time steps 0.2 and 0.1 fs, respectively, at 300 K to ensure a fully converged system for the production run. The production runs for simulation were carried out at a constant temperature of 300 K and a pressure of 1 atm or bar (NPT) using weak coupling velocity-rescaling (modified Berendsen thermostat) and Parrinello-Rahman algorithms, respectively. All bond lengths involving hydrogen atoms were kept rigid at ideal bond lengths using the Linear Constraint Solver (lincs) algorithm, allowing for a time step of 2 fs. The Verlet scheme was used for the calculation of non-bonded interactions. Periodic Boundary Conditions (PBC) were used in all x, y, and z directions. Interactions within a short-range cutoff of 1.2 nm were calculated in each time step. Particle Mesh Ewald (PME) was used to calculate the electrostatic interactions and forces to account for a homogeneous medium outside the long-range cutoff.

### Epitope-omics: MELD/LC–MS

#### Multi-enzymatic-limited-digestion (MELD)

In this experiment, MELD was used to prepare the immunocomplexes for peptide mapping in epitope analysis. In brief, an anti-CTX monoclonal antibody (TPL–27_01_F7 scFv clone, Absolute Antibody, United Kingdom), was diluted according to the ratio of 1:5000, 1:10,000, and 1:30,000 in phosphate-buffered saline (PBS). The anti-CTX was mixed with pure CTX (see section “Expression of CTX^WT^ and CTX^VAR^”) *in* 1:1 (v/v) for 2 h at room temperature to form immunocomplexes. Subsequently, the immunocomplexes were concentrated in PBS, and the unbound CTX was removed with a 10 kDa Amicon^Ⓡ^ Ultra Centrifugal Filter Spin Column (Merck, United States). The immunocomplex was then reduced with dithiothreitol (DTT; Thermo Fisher Scientific, United States) and alkylated and iodoacetamide (IAM; Thermo Fisher Scientific, United States). This was followed by proteolytic digestion with multiple endoproteases. The endoproteases used in MELD were trypsin (Thermo Fisher Scientific, United States), chymotrypsin (Thermo Fisher Scientific, United States), and Lys-C (New England Biolabs, United States) in a 1:20 enzyme-to-protein ratio by mass. The reaction mixtures were incubated at 37 °C overnight before desalting using ZipTip^Ⓡ^ pipette tips (Merck, United States). The digested peptides were then vacuum-dried before LC–MS analysis.

#### Examination of immunocomplex formation by immunoturbidimetric assay

The immunoturbidimetric assay protocol was modified from a reported study by O’ Leary and colleagues^60^. Briefly, a two-fold serial dilution of anti-CTX from 50 µg/mL in 50 µL PBS was added into a 96-well plate. Then, another 50 µL of CTX (25 µg/mL) was added into each well, resulting in a final concentration of 12.5 µg/mL. Absorbance readings were taken every minute at wavelength 340 nm using the Tecan microplate plate reader at 37 °C for 40 min. The kinetics of the immunocomplexes formation were then plotted and examined.

#### Liquid chromatography-mass spectrometry (LC–MS)

The identification of the MELD-peptides was conducted using the Agilent 1200 HPLC-Chip/MS Interface, coupled with Agilent 6550 iFunnel Q-TOF LC/MS instruments. The lyophilized samples were reconstituted in solvent A (0.1% formic acid in water). Subsequently, 1 µL of the sample was injected into the C18 enrichment and analytical column (Agilent Large Capacity Chip, 300 Å, C18, 160nL enrichment column & 75μm × 150mm analytical column) at a flow rate of 4 µL/min from Agilent 1200 Series Capillary pump and 0.5 µL/min from Agilent 1200 Series Nano Pump with solvent B (90% of acetonitrile in water with 0.1% formic acid). The peptide mixtures were separated for 50 min under an increasing gradient of solvent B from 5 to 70%, followed by an 8-min post-run analysis. The elution of peptides was configured as follows: positive ion mode, capillary voltage 1.9 kV, fragmentor voltage 360 V, gas temperature 325 °C, drying gas flow 5.0 L/min. Mass spectra were acquired in an MS/MS mode over a mass (*m/z*) range of 50–3000. Solvent A was used as a blank to eliminate peptide contamination from other sources. The data was then processed using the Peaks X + software, searching against the Serpentes protein database (Taxid: 8570).

### Multiple sequence alignment

To finalize the potential epitope sequences of CTX, multiple sequence alignment of predicted epitopes was performed using the Clustal Omega^49–51^ and visualized using Jalview 2.11.1.3^81^. Several parameters, such as overlapping sequences, degree of sequence conservation, and the number of amino acids residing within functional sites of CTX, were considered.

### Functional epitope site mapping of cytotoxin by site-directed mutagenesis

To further confirm the presence of epitopes at the functional loops of CTX, functional epitope mapping of CTX was performed by site-directed mutagenesis.

#### Preparation of pET-22b ( +)-CTX^WT^ and CTX^VAR^

Polymerase chain assembly (PCA) was used to synthesize wildtype CTX (CTX^WT^) and variant (CTX^VAR^) genes by Economy Gene Synthesis (GenScript, United States). Both genes were inserted into pET-22b ( +) plasmids at the NdeI/XhoI restriction sites (Supplementary File-Fig. S3), respectively, and transformed into *Escherichia coli* Dh10B cells for plasmid expansion.

#### Expression of CTX^WT^ and CTX^VAR^

The pET-22b ( +)-CTX^WT^ and CTX^VAR^ plasmids were extracted from glycerol stock of DH10B cells using GF-1 Plasmid DNA Extraction kit (Vivantis, Malaysia) following the manufacturer’s protocol. The CTX^WT^ and CTX^VAR^ genes were amplified by touch-down PCR with their respective forward and reverse primers (Supplementary File 1-Table S2).

To express CTX^WT^ and CTX^VAR^, pET-22b ( +) plasmids harboring the respective genes were first transcribed into RNA using the HiScribe™ T7 Quick High Yield RNA Synthesis Kit (New England Biolabs, United States). The transcribed RNAs were then expressed using NEBExpress® Cell-free *E. coli* Protein Synthesis System (New England Biolabs, United States) for in vitro protein translation, according to the manufacturer's protocol. After that, CTX^WT^ and CTX^VAR^ were purified using a HisTrap™ HP, 1 mL affinity column on ÄKTA Pure protein purification system (Cytiva, United States). The HisTrap column was equilibrated with buffer A (20 mM sodium phosphate, 0.5 M NaCl, pH 8.0) before the samples were injected into the column. CTX^WT^ and CTX^VAR^ were purified with a gradient elution from 4 to 100% of buffer B (20 mM sodium phosphate, 0.5 M NaCl, 500 mM imidazole, pH 8.0) at a flow rate of 500 uL/min. The presence of his-tagged expressed CTX and its purity were visualized by western blot.

#### Cytotoxicity of CTX^WT^ and CTX^VAR^

The cytotoxicity of CTX^WT^ and CTX^VAR^ was determined using the cell-counting kit-8 (CCK-8) assay (Sigma Aldrich, United States). Briefly, 100,000 cells/mL of human skin keratinocytes, Hacat cell lines (Elabscience Biotechnology Inc, United States), were seeded in a 96-well plate at 37 °C, 5% CO_2_ overnight. The cells were treated with 100 µL of 8 µg/mL CTX^WT^ and CTX^VAR^ in serum-free cell culture media for 24 h. Ten microliters of CCK-8 reagent were added to each well and incubated for 1 h. The level of cell viability was quantified through the formation of orange-colored products upon reduction of the WST-8 salts present in the CCK-8 reagent by dehydrogenase in viable cells. The absorbance readings were obtained using the Tecan microplate reader at wavelength 450 nm. The percentage (%) of cell viability was calculated.

#### Homology modeling of CTX^WT^ and CTX^VAR^

We performed homology modeling on MODELLER 9.21^82^ to determine if site-directed mutagenesis produced any structural changes to CTX^VAR^. The DOPE per residue profile of each amino acid residue within the CTX^WT^ and CTX^VAR^ was plotted on Gnumeric for comparison. CTX^WT^ and CTX^VAR^ models were subjected to further visual evaluation using BIOVIA Discovery Studio Visualizer 2021 by superimposing both models. The corresponding root-mean-squared deviation (RMSD) value was also determined for CTX^WT^ and CTX^VAR^ models.

## Conclusion

Potential T-cell and B-cell epitopes of CTX were identified to be 'KLVPLFY,' 'CPAGKNLCY,' 'MFMVSTPTKVP,' and 'DVCPKNSLL’, whereby 'KLVPLFY,' ‘MFMVSTPTKVP,' and 'DVCPKNSLL’ were found to reside within the functional loops of CTX. 'CPAGKNLCY' constituted the region between loops I and II, which is vital for CTX’s structural folding and stability. HLA-B62 supertypes was identified to possess the highest binding towards CTX. The intermolecular bonds between the HLA-CTX complex were hydrogen bonds, *Van der Waals* interactions, and salt bridges. The crucial amino acid residues on CTX, namely L7, G18, K19, N20, M25, K33, V43, C44, K46, N47, and S48 were involved in intermolecular interactions with HLA-B62. The epitope-omic analysis revealed three highly potential epitope sequences ' LVPLFYK,' 'MFMVS,' and ‘TVPVKR,’ which were also located at the functional loops I and II. Different mapping approaches finalized the potential epitopes of CTX as ‘KLVPLFYK’, ‘AGKNL’, ‘MFMVSTPKVPV’ and ‘DVCPKNSLL.’ Furthermore, site-directed mutagenesis of these four epitope sites altered the physicochemical properties and cytotoxicity of CTX albeit the overall structure of CTX was retained. This concluded that, the identified epitope sequences were located at the functional loops of CTX. Since the functional loops of CTX form its hydrophobic three-finger folded core, our findings provide a justification of low immunogenicity of CTX, which results in inadequate efficacy of existing antivenoms targeting CTX. In conclusion, the CTX’s functional epitopes represented consensus epitope properties of different CTX isoforms. These functional epitopes can be fabricated as synthetic epitopes to produce toxin-targeted cross-reactive antivenom that antagonize the cytotoxic effects in dermonecrosis.

## Supplementary Information

Supplementary Information.

## Data Availability

The mass spectrometry proteomics datasets generated and/or analyzed during the current study are available in the ProteomeXchange Consortium (https://www.proteomexchange.org/) via the PRIDE partner repository with the dataset identifier PXD040420.
