# Supplementary material for: Unveiling the functional epitopes of cobra venom cytotoxin by immunoinformatics and epitope-omic analyses
Source: Sci Rep. 2023 Jul 28;13:12271. doi: 10.1038/s41598-023-39222-2 (PMC10382524; doi:10.1038/s41598-023-39222-2)
Supplement: Supplementary file 1 — Supplementary Information. [file 41598_2023_39222_MOESM1_ESM.docx]

**Unveiling the functional epitopes of cobra venom cytotoxin by immunoinformatics and epitope-omic analyses**

Hiu Jia Jin, Fung Jared Kah Yin, Tan Hock Siew, Yap Michelle Khai Khun*

School of Science, Monash University Malaysia, Bandar Sunway, 47500 Malaysia

*Corresponding author: Yap Michelle Khai Khun

Email: [yap.michelle@monash.edu](mailto:yap.michelle@monash.edu)

Tel: +603-55145860


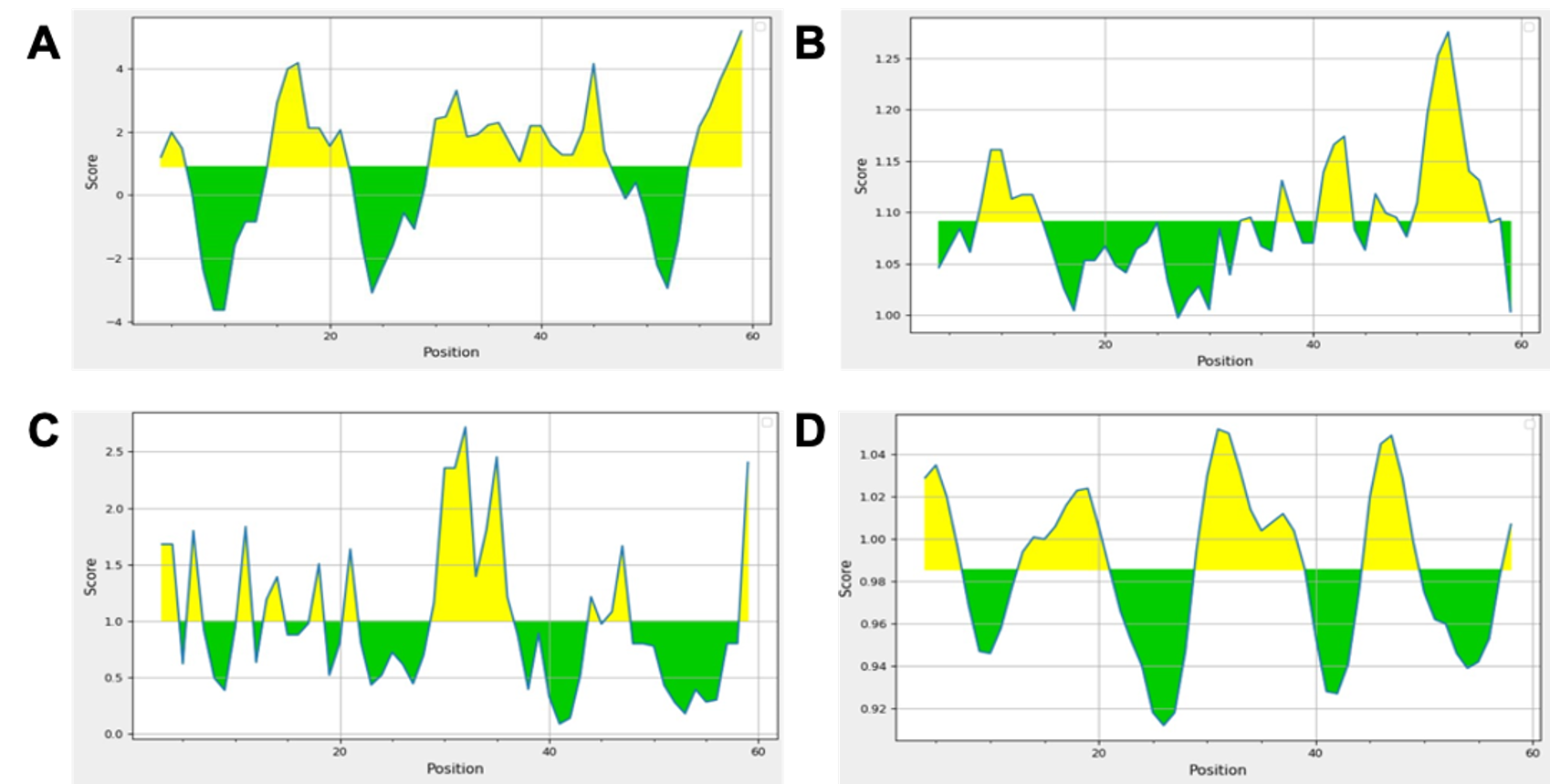


**Figure S1**. Graphical representation of different B-cell epitope prediction parameters. The threshold value of each prediction parameter is annotated as a red line. Yellow peak areas above the threshold were predicted as B-cell epitopes, while green peak areas below the threshold were not B-cell epitopes. A, Parker hydrophilicity prediction, the threshold value was 0.895. B, Kolaskar & Tongaonkar antigenicity prediction, the threshold value was 1.091. C, Emini surface accessibility prediction, the threshold value was 1.000. D, Karplus & Schulz's flexibility prediction, the threshold value was 0.986.


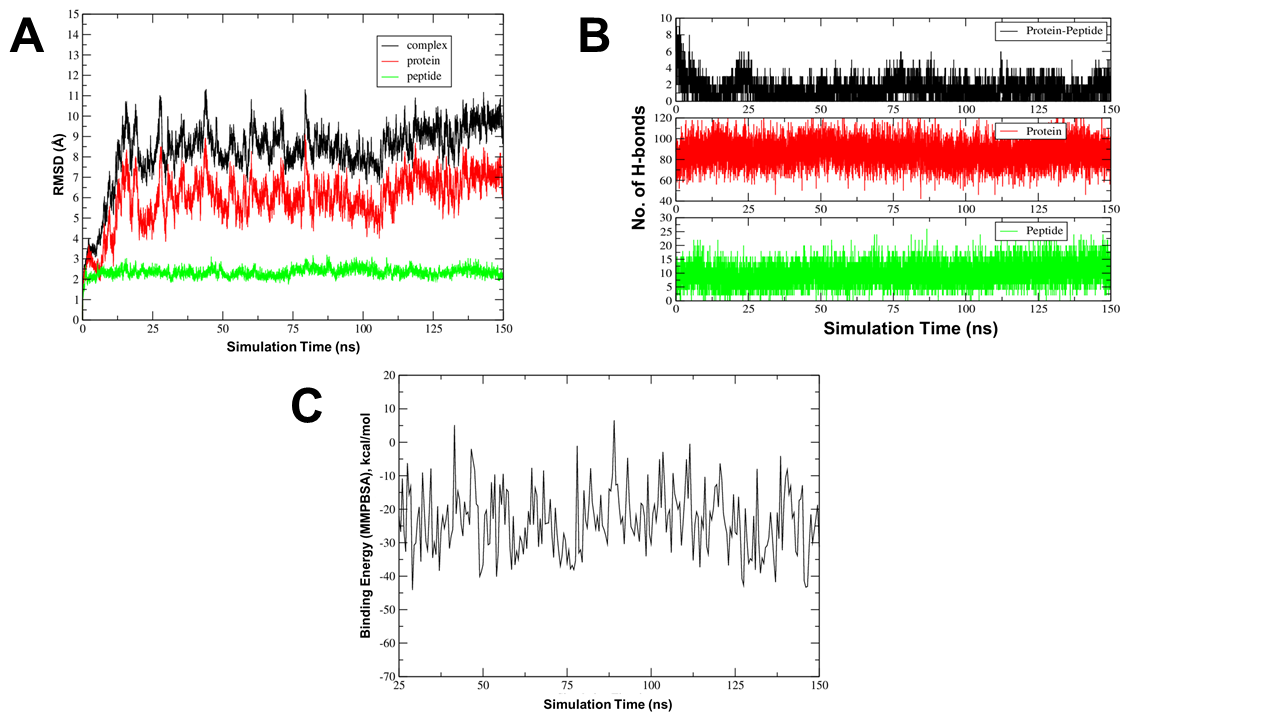


**Figure S2**. Molecular dynamic simulations of HLA-B62-CTX complex. A, Mean RMSD value calculated based on C-α using Bio3D program of R, for 125 ns, the mean RMSD values for docking complex were 8.73 ± 0.80 Å. B, Total number of hydrogen bonds formed between protein-peptide complex, within protein and peptide during 150ns of simulation, the mean H-bond numbers between protein-peptide were 1.56 ± 0.99. C, Binding energy of protein-peptide complex obtained by MMPBSA method of the MD trajectory. The mean MMPBSA binding energy was found to be -23.55 ± 9.71 kcal/mol.


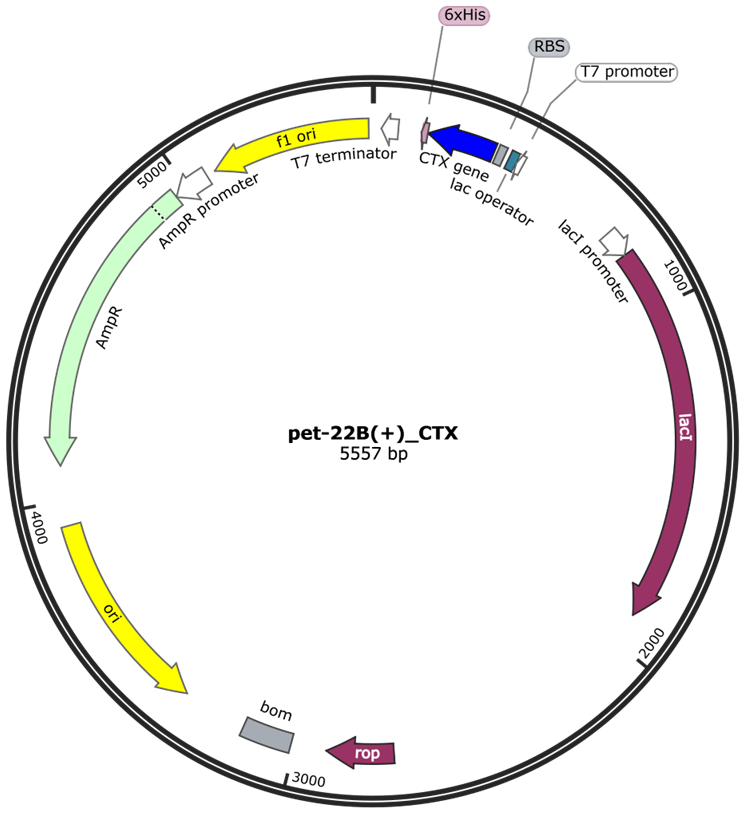


**Figure S3**. Vector map of the pET-22b (+)-CTX^WT^ and CTX^VAR^. The ‘CTX gene’ presented in the vector (in blue) encompassed both sequences of CTX^WT^ and CTX^VAR^, respectively.





**Figure S4**. The mass spectra of the representative peptides identified from MELD-LC/MS of 1:5000 anti-CTX immunocomplexes.

**Table S1.** Assignment of peptides list identified from MELD-LC/MS analysis at different dilution of anti-CTX

| **Dilution factor** | **Peptide Sequence** | **-10lgP** | **Mass** | ***m/z*** | **Accession No.** |
| --- | --- | --- | --- | --- | --- |
| 1:5,000 | EIVDLHNSLR | 35.3 | 1194.636 | 399.2176 | P0DL15, P0DL16, P86543, A0A8C7E3T9, A0A6J1VUF3, F2Q6G0, F2Q6E6, F2Q6E8, F2Q6E9, F2Q6E7, F2Q6E5, F2Q6G1, V8N8B4, P60623, A0A8C6XXU9, A0A223PK48, P84805, A0A8C6XXV5, Q7ZT98, F2Q6F2, Q7T1K6, Q8JI40, F2Q6F7, A0A6B2F4F1, F2Q6E4, A0A2H4N3D5, T2HPR8, F2Q6E3, A0A0K8S072, A0A1L8D673, A0A223PK22 |
|  | MFMMSDLTIPVKR | 60.86 | 1599.782 | 534.2725 | Q91136, P60304, Q91135, P79810, Q98958, Q9PS33, P01451 |
|  | MFMMSDLTIPVK | 53.33 | 1427.686 | 714.8521 | Q91136, P60304, Q91135, P79810, Q98958, Q9PS33, P01451 |
|  | LIPIASK | 50.44 | 768.4745 | 385.2426 | Q91136, P60304, Q91135, P79810, Q98958 |
|  | MFMVATPK | 41.33 | 923.4609 | 462.7393 | Q98960, P60302, Q98959, Q02454, P60301, E2ITZ7, P60303, Q91124, Q98962, P60307, P60308 |
|  | MFMVSNLTVPVKR | 39.62 | 1520.821 | 507.9476 | O73857, P01442, P01443, Q9W6W9, P60310 |
|  | MFMVSNLTVPVK | 39.26 | 1364.72 | 683.3686 | O73857, P01442, P01443, Q9W6W9, P60310 |
|  | LTCLICPEK | 36.17 | 1132.562 | 567.2878 | P0DSM9, P29179, P29180, P01401, A0A8C6XDU5, P85520, P01400, D5J9Q0, Q8AY50, D5J9Q1, A2CKF7, Q8AY49, E2ITZ6, O93422, D5J9P9, Q6IZ95, Q9YGI1, Q8AY51, Q9YGI2 |
|  | MSNIVTCQPWEK | 35.89 | 1491.685 | 746.8477 | P0DSN0 |
|  | ETIYSFAK | 32.48 | 957.4807 | 479.7503 | - |
|  | ETLYSFAK | 32.48 | 957.4807 | 479.7503 | P0CB14 |
|  | LVPLFYK | 31.84 | 906.5214 | 454.2695 | O73857, P01442, P01443, Q9W6W9, P60310 |
|  | GFPGTVGPK | 29.83 | 858.4599 | 430.2213 | A0A6I9YM71, A0A6I9YN95, A0A6P9CIW2, A0A6P9CEP9, A0A6P9CTZ8, A0A6I9YML1, A0A6I9YLH8, A0A6I9YLW8, A0A6I9YM65, A0A6I9YN87, A0A6P9CT09, A0A6P9CIV6, A0A6P9CEP3, A0A6P9CHU9, A0A6P9CTZ1, A0A6P9CT04, A0A6P9CIV1, A0A6P9CHU5, A0A6P9CEN7, A0A6P9CTX9 |
|  | MFMVSNK | 26.6 | 855.3983 | 428.7066 | P0C944, P07525, P0CH80, O73858, Q98961, P24780, P01446, P01445, A0A0U4N5W4, A0A8C6XLA1 |
|  | MYMVAMPK | 25.84 | 969.4486 | 485.7341 | O73856 |
|  | VYLVIVADK | 25.77 | 1018.606 | 510.3125 | P0CB14 |
| 1:10,000 | MFMVSNLTVPVKR | 45.03 | 1520.821 | 507.9458 | P60310, Q9W6W9, P01443, P01442, O73857 |
|  | MFMMSDLTIPVKR | 39.03 | 1567.793 | 523.6044 | Q9PS33, P01451 |
|  | MFMMSDLTIPVK | 38.49 | 1411.691 | 706.8522 | Q9PS33, P01451 |
|  | MFMVSNLTVPVK | 36.73 | 1364.72 | 683.368 | P60310, Q9W6W9, P01443, P01442, O73857 |
|  | LIPLAYK | 31.62 | 816.5109 | 409.2643 | A0A0U5AR60 |
|  | MFMVATPK | 30.06 | 923.4609 | 462.7368 | P60303, E2ITZ7, P60301, Q02454, Q98959, P60302, Q98960 |
|  | LVPIAYK | 29.78 | 816.5109 | 409.2643 | Q9PS33, P01451 |
|  | LVPLFYK | 29.52 | 878.5266 | 440.2705 | P60310, Q9W6W9, P01443, P01442, O73857, P60303, E2ITZ7, P60301, Q02454, Q98959, P60302, Q98960 |
| 1:30,000 | MFMVSNLTVPVKR | 51.18 | 1520.821 | 507.9514 | O73857, P01442, P01443, Q9W6W9, P60310 |
|  | MFMMSDLTIPVK | 46.28 | 1411.691 | 706.8501 | Q9PS33, P01451, P60305, E2IU04, Q91136, P60304, Q91135, Q98957, P79810, Q98958 |
|  | MFMMSDLTIPVKR | 45.45 | 1567.793 | 523.6053 | Q9PS33, P01451, P60305, E2IU04, Q91136, P60304, Q91135, Q98957, P79810, Q98958 |
|  | MFMVATPK | 36.91 | 923.4609 | 462.7375 | Q98960, P60302, Q98959, Q02454, P60301, E2ITZ7, P60303, P60307, P60308, Q91124, Q98962 |
|  | MFMVSNLTVPVK | 31.61 | 1364.72 | 683.3646 | O73857, P01442, P01443, Q9W6W9, P60310 |

**Table S2.** Touchdown PCR conditions

| **Stage** | **Temperature (°C)** | **Time (min)** | **Cycles** |
| --- | --- | --- | --- |
| Initial Denaturation | 94.0 | 03:00 | 1 |
| Denaturation | 94.0 | 00:30 | 30 |
| Annealing | 66-56 (CTX^WT^)  65-55 (CTX^VAR^) | 00:30 | 30 |
| Extension | 72.0 | 00:30 | 30 |
| Initial Denaturation | 94.0 | 00:30 | 1 |
| Denaturation | 56 (CTX^WT^)  55 (CTX^VAR^) | 00:30 | 1 |
| Annealing | 72.0 | 00:30 | 1 |
| Extension | 72.0 | 10:00 | 1 |
| Rest | 4.0 | ꝏ | ꝏ |

Forward primer for CTX^WT^:

5’-GCGAATTAATACGACTCACTATAGGGCTTAAGTATAAGGAGGAAAAAATATGCTGAAATGCAACAACAAACTGGT-3’

Forward primer for CTX^VAR^:

5’-GCGAATTAATACGACTCACTATAGGGCTTAAGTATAAGGAGGAAAAAATATGCTGAAGTGCAATAATAAACTGGT-3’

Reverse primer for both CTX^WT^ and CTX^VAR^:

5’-AAACCCCTCCGTTTAGAGAGGGGTTATGCTAGTTAGTGGTGGTGGTGGTGGTG-3’
